# Drastic Events and Gradual Change Define the Structure of an Active Copper‐Zinc‐Alumina Catalyst for Methanol Synthesis

**DOI:** 10.1002/anie.202200301

**Published:** 2022-03-02

**Authors:** Arik Beck, Mark A. Newton, Maxim Zabilskiy, Przemyslaw Rzepka, Marc G. Willinger, Jeroen A. van Bokhoven

**Affiliations:** ^1^ Institute for Chemistry and Bioengineering ETH Zurich Vladimir-Prelog-Weg 1 8093 Zürich Switzerland; ^2^ Laboratory for Catalysis and Sustainable Chemistry Paul Scherrer Institute Forschungsstrasse 111 5232 Villigen Switzerland; ^3^ Scientific Center for Optical and Electron Microscopy (ScopeM) ETH Zurich Otto-Stern-Weg 3 8093 Zürich Switzerland

**Keywords:** Activation, CO_2_ Conversion, Copper, *operando* Analysis, Zinc

## Abstract

The copper‐zinc‐alumina (CZA) catalyst is one of the most important catalysts. Nevertheless, understanding of the complex CZA structure is still limited and hampers further optimization. Critical to the production of a highly active and stable catalyst are optimal start‐up procedures in hydrogen. Here, by employing *operando* X‐ray absorption spectroscopy and X‐ray diffraction, we follow how the industrial CZA precursor evolves into the working catalyst. Two major events in the activation drastically alter the copper‐ and zinc‐containing components in the CZA catalyst and define the final working catalyst structure: the reduction of the starting copper(II) oxide, and the ripening and re‐oxidation of zinc oxide upon the switch to catalytic conditions. These drastic events are also accompanied by other gradual, structural changes. Understanding what happens during these events is key to develop tailored start‐up protocols that are aimed at maximal longevity and activity of the catalysts.

## Graphical abstract

The origin of the high performance of the extensively used copper‐zinc‐alumina (CZA) catalyst for methanol synthesis is still unknown. How the catalyst is treated before use strongly determines its ultimate performance. Here, using combined *operando* X‐ray methods the evolution of the industrial CZA catalysts is followed and key events that define the final structure are identified.

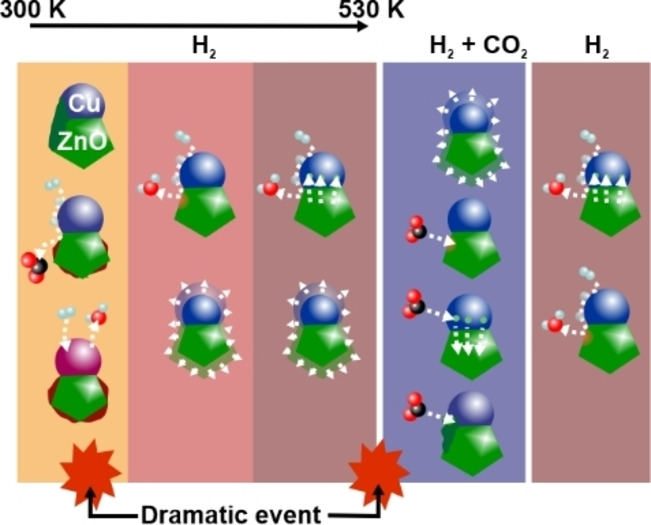

## Introduction

Since its inception in the 1920′s,[^1^, ^2^, ^3^, ^4^, ^5^, ^6^] the copper‐zinc‐alumina (CZA) catalyst formulation has remained the most effective, and industrially preferred, solution for the bulk production of methanol from syngas.[^7^, ^8^] Recently, CZA is intensively researched as a leading contender for the commercial and direct hydrogenation of carbon dioxide.[^9^, ^10^, ^11^, ^12^] However, in both applications, the details of the superior performance of this multicomponent catalyst have remained elusive. Numerous hypotheses exist to explain the superior behaviour in methanol synthesis, including: the apparent proportionality between copper surface area and activity;[^13^, ^14^] the role of the formation of an α‐brass, CuZn alloy phase, or Zn^0^ species decoration of the copper surface;[^15^, ^16^, ^17^, ^18^, ^19^, ^20^, ^21^, ^22^, ^23^, ^24^, ^25^] the influence of oxygen vacancies within the zinc oxide, whose presence may be promoted by the formation of metal/semiconductor interfaces in the active catalyst;[^26^, ^27^] and, the possibility that defects in the copper,[^19^, ^28^] or Cu^I^ sites,^[7]^ are responsible for the exceptional activity characteristics of this formulation. A strong dependence of structure on the conditions has been established, and the existence of pressure‐ and material‐gaps is at the origin of this panoply of partially contradicting theories.[^29^, ^30^]

Despite this ongoing debate, there is a consensus that a high copper surface area, and its intimate contact with zinc species, are key to an active and stable catalyst.^[31]^ Any industrial CZA catalyst has to expose the maximum amount of the relevant structural elements during the steady state operation of the catalyst. Here, the start‐up procedure is critical. It includes reduction of the catalyst precursor in hydrogen and initial contact with the catalytic reaction mixture. An unsuitable start‐up protocol may have drastic consequences for the catalyst activity and lifetime. Therefore, it is essential to understand the structural details of catalyst's transformations in this process in order to tailor matching start‐up protocols.^[32]^ In this work, we illustrate how the CZA catalyst undergoes both gradual and sudden structural changes during reductive activation and switching to reactive conditions. In situ, time‐resolved Cu and Zn K‐edge X‐ray absorption near‐edge spectroscopy (XANES), extended X‐ray absorption fine structure (EXAFS), and X‐ray powder diffraction (XRD) identify the complex interactions and synergisms between the copper and zinc components which characterize industrial CZA systems.

## Results and Discussion

**Figure 1:**
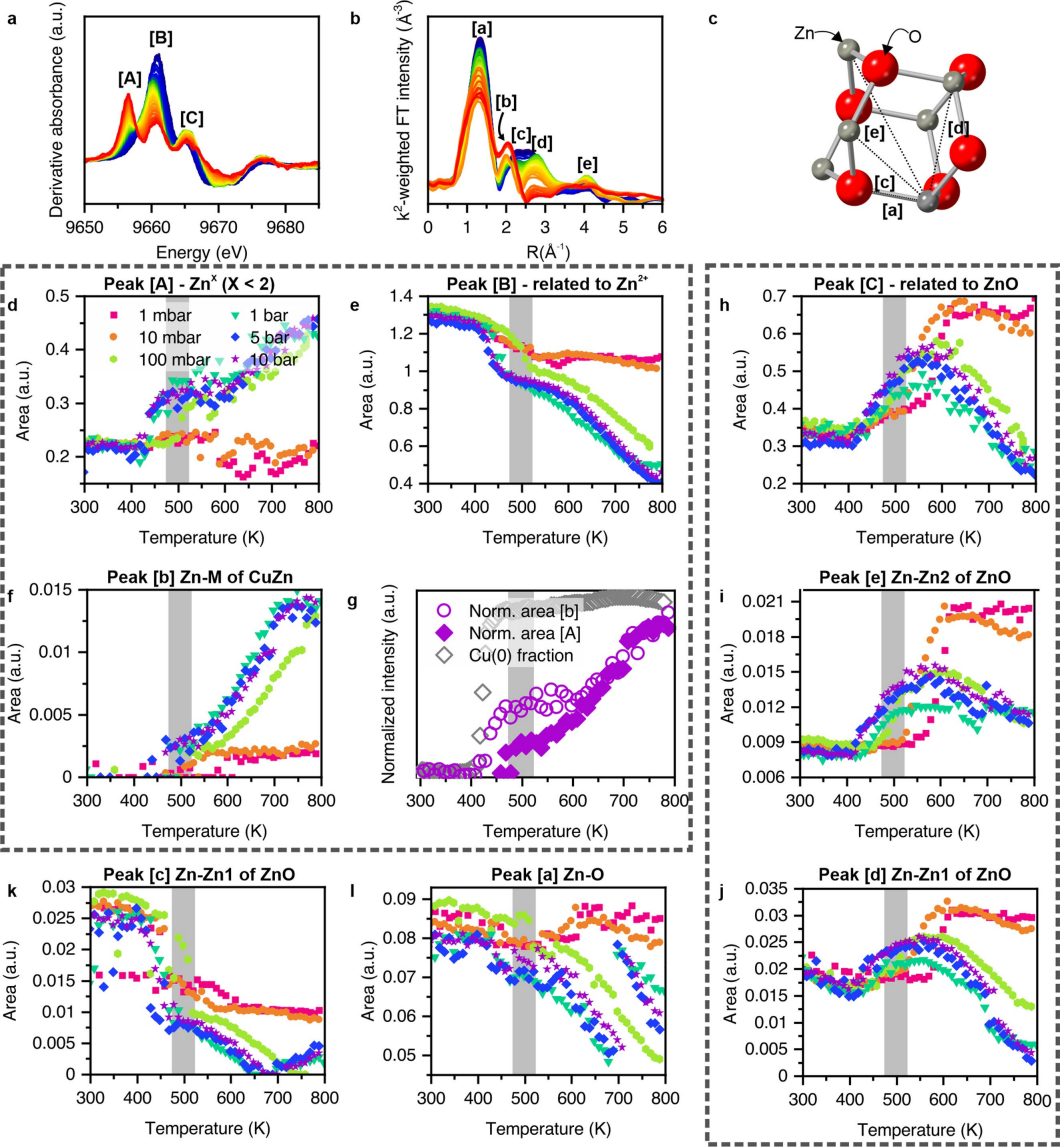
a) First derivative normalized Zn K‐edge XANES and b) non‐phase corrected Fourier transform representations of the k^2^‐weighted Zn K‐edge EXAFS obtained during temperature‐programed reduction (TPR) of the CZA catalyst to 800 K at 10 bar. c) Their respective correspondence to scattering distances in *hcp* zinc oxide (Feature [b] can be associated to the Zn−Cu scattering in an *fcc* CuZn alloy). d), e), h) Temperature‐dependent behaviour of three features ([A]–[C]) isolated from the derivative Zn K‐edge XANES shown in a, for a range (1 mbar≤*p*(H_2_)≤10 bar), of applied hydrogen pressure during reduction to 800 K. f), i), k), l), j) Peak areas derived from Fourier transforms of k^2^‐weighted Zn K‐edge EXAFS for the five features as a function of temperature and applied hydrogen pressure during temperature programmed reduction to 800 K. g) Evolution of the area of the XANES feature [A] and the EXAFS feature [b] which both are directly linked to the reduction of Zn^II^. Feature [b] is directly linked to the evolution of a Cu−Zn scattering pair due to alloy formation. Also shown is the Cu^0^ fraction determined by linear combination fittings see ref. [24].

In an industrial activation protocol low hydrogen concentrations (*p*(H_2_)<100 mbar) are initially employed, and only after a reduction at these pressures, the hydrogen pressure is stepwise increased.[^33^, ^34^] Consequently, it is very important to understand how the structure develops during activation at different hydrogen pressures. The evolution of the Cu K‐edge XANES and its interpretation in terms of transformation of the copper phase measured in the same experiment can be found in ref. [29]. It was found that the reduction of Cu^II^ to metallic copper in hydrogen is a strong function of pressure. At 1 mbar, the reduction event occurs at around 573 K. When the hydrogen pressure is increased, the temperature at which Cu^II^ reduced continuously shifts to lower temperatures. At pressures of 5 bar and above, the reduction occurs at 423 K.^[29]^ Figure 1a and b show the development of Zn K‐edge XANES and non‐phase corrected Fourier transformed (FT) EXAFS observed during in situ temperature‐programmed reduction (TPR) of the CZA catalyst under 10 bar of hydrogen up to 800 K.^[29]^ Similar plots for the other pressures studied can be found in Supplementary Figure S1. Within the derivative XANES, at least three relevant peaks change (here labelled as [A], [B], and [C]). By comparison, we can evaluate the presence of these XANES features within our reference components (Table 1), as identified in ref. [29]. The pre‐edge feature [A] is present in spectra of both metallic zinc and in a copper‐zinc alloy, and the feature [B] in XANES of the zinc components having an oxidation state +2 and the precursor state zinc carbonate.[^29^, ^35^, ^36^] Feature [C] is mainly present in the XANES of *hcp* zinc oxide. Within the FT‐EXAFS, five peaks are seen to change during the course of the heating (labelled [a]–[e]). The main scattering path contribution to each of the FT‐EXAFS features can be obtained by EXAFS analysis (Table 2, Supplementary Figure S1, Figure 1c). Features [a], [c], [d], and [e] can be assigned to the scattering within the *hcp* zinc oxide structure. Feature [b], however, is due to Zn−Cu scattering interactions, and indicative of the formation of Zn^0^ and of an *fcc* CuZn, α‐brass phase. The evolution of the derivative XANES and EXAFS at five different hydrogen pressures (1 mbar–10 bar) were evaluated using a Gaussian peak deconvolution (Supplementary Figure S3). Figure 1d–k shows the development of the peak areas with respect to hydrogen pressure and temperature. By cross‐referencing the development of the various peaks, the features can be grouped into ones of similar behaviour (dashed boxes in Figure 1): Features [A] and [b] develop together, while [B] appears inversely correlated to these. Above 100 mbar, however, [A] and [B] undergo a stepwise in‐/decrease of ca. 40 % in intensity at around 400 K, while [b] starts to increase in a monotonous manner at higher temperatures. This is best depicted by the comparison of the normalized evolution of [A] and [b] and the evolution of the fraction of metallic copper determined by linear combination fitting, which starts to appear at 400 K in 10 bar of hydrogen (Figure 1g). In the XANES, the formation of Zn^0^ is expected to result in a lower binding energy feature. However, the observation of such a XANES feature is not an unequivocal indicator for the presence of the CuZn phase,[^37^, ^38^, ^39^] and it is evident from the behaviour of this feature, at *p*(H_2_)≥100 mbar, that more than one process is contributing to it. Definitive evidence, from the appearance of [b] in the EXAFS, for the production of a CuZn phase is only observed above 470 K. The step in feature [A] occurs at the point wherein the copper component is reduced from copper(II) oxide to metallic copper nanoparticles. In a second group, features [C], [d], and [e] develop in a similar manner (Figure 1h–j). EXAFS features [d] and [e] are assigned to first shell and second shell Zn−Zn scattering of the *hcp* zinc oxide structure. At the onset temperature of copper reduction, all three features increase in peak area. Below 100 mbar of hydrogen pressure, the features increase up to 600 K and then remain at a constant intensity. At 100 mbar and above, the features start to diminish again at 550 K. In comparison, features [c] and [a] (Figure 1k, l), which also both come from the first shell scattering (Zn−Zn and Zn−O) of the *hcp* zinc oxide structure, behave differently. Both signals decrease with rising temperature. The desynchronization of several scattering features which belong to the same structure suggest an anisotropic increase of order in the zinc oxide. At temperatures beyond 550 K, this order starts to disappear again.

**Table 1:** Interpretation of the XANES features.

XANES Features	Energy [eV]^[a]^	Present in spectrum of reference component^[b]^
[A]	9656.4–9657.6	Zn0, CuZn
[B]	9660–9660.9	ZnO, ZnCO3(H_2_O)_*X*_
[C]	9664.9–9665.5	ZnO

[a] Observed shift of the peak energy position within the TPR experiment. [b] Supplementary Figure S4 depicts selected reference for Zn^0^, CuZn, ZnO, and ZnCO_3_(H_2_O)_*X*_. Assignment is based on the presence of the peak in these spectra.

**Table 2:** Interpretation of the EXAFS features.

EXAFS Features	Non‐phase corrected Radial position [Å]	Assignment
[a]	1.32	1^st^ shell Zn−O
[b]	2.02	1^st^ shell Zn−Cu (of *fcc* CuZn)
[c]	2.27	1^st^ shell Zn−Zn (of ZnO)
[d]	2.77	1^st^ shell Zn−Zn (of ZnO)
[e]	4.081	2^nd^ shell Zn−Zn (of ZnO)

**Figure 2:**
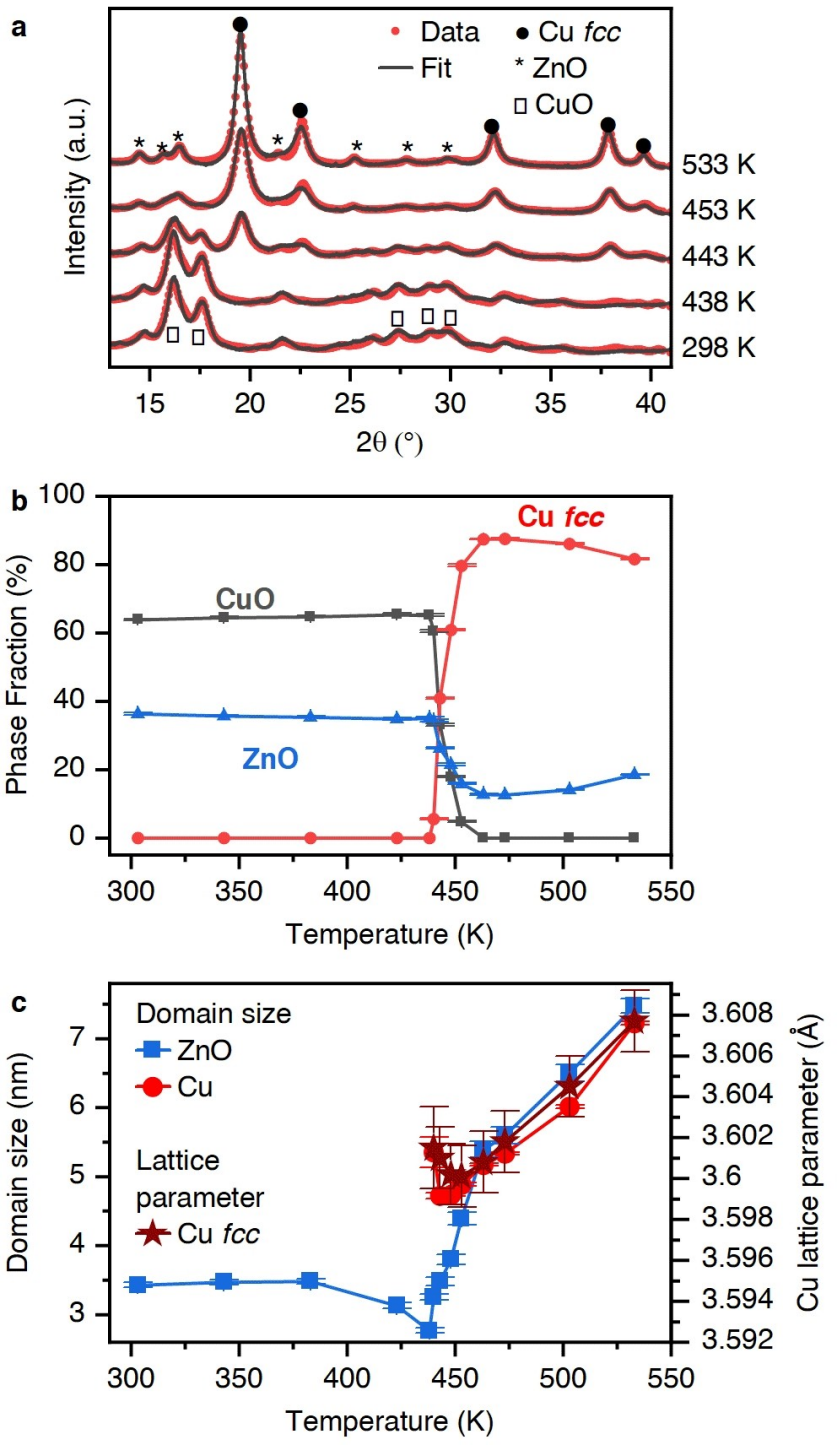
a) Representative XRD patterns collected as a function of temperature during in situ reduction of the CZA catalyst to 533 K under 12 bar hydrogen. The position of Bragg reflections due to CuO, ZnO (wurtzite) and an *fcc* phase are also indicated. b) Rietveld analysis derived evolution of ZnO, CuO, and Cu *fcc* fractions of the three phases; and c) the corresponding domain sizes as a function of temperature; as well as the evolution of the Cu *fcc* lattice parameter.

XANES and EXAFS, however, remain limited in their ability to describe the specifics of how the copper and zinc oxide structures evolve beyond a size of abound 5 nm. We therefore performed time‐resolved in situ XRD. Figure 2, and Supplementary Figure S5, shows XRD data obtained during in situ reduction of a CZA catalyst under 12 bar hydrogen, from ambient temperature to 533 K. All XRD patterns of the CZA catalysts were matched to structures of copper(II) oxide, *hcp* zinc oxide, and an *fcc* phase. The latter can either represent copper or α‐brass (Figure 2a). The presence of alumina structure is not represented by Bragg reflections, neither ZnAl_2_O_4_ spinel structure could be detected at any stage. Therefore, the role of alumina in the structural evolution is not discussed within this work, even though it may play an essential role in the formation of the final structure.[^36^, ^40^, ^41^] Figure 2b shows the phase composition, which arises from Rietveld analysis, as a function of temperature. The transition between copper(II) oxide and its reduced *fcc* counterpart at 435 K is sharp. This temperature marks the point upon which several severe changes in the data occur. The careful refinement enabled the determination of domain sizes and lattice parameters of the crystallites (Figure 2c). The average zinc oxide domain size remains around 3.5 nm up to 435 K and then grows rapidly reaching 5.5 nm at 470 K. Beyond this temperature the growth is slower and the domains have grown to 7.5 nm by the time the catalyst bed reaches 530 K. The initial mean copper domain size at 435 K was 5.2 nm. The domain size remains at this value up to 470 K, at which point, the domain size increases in the same, correlated, fashion observed for zinc oxide to also reach an average size of 7.5 nm. Also, at 435 K, an apparent loss of the ordered zinc oxide phase visible to Bragg diffraction occurs (Figure 2b). Below this temperature, the zinc oxide phase accounted to around 35 % of the crystal phase composition. When the TPR experiment reached 470 K, the XRD visible zinc oxide phase content dropped to 12 %. The growth of the *fcc* phase is accompanied by a parallel, linear with temperature, expansion of the lattice parameter toward, but does not attain, the expected bulk copper lattice constant (Figure 2c).

**Figure 3:**
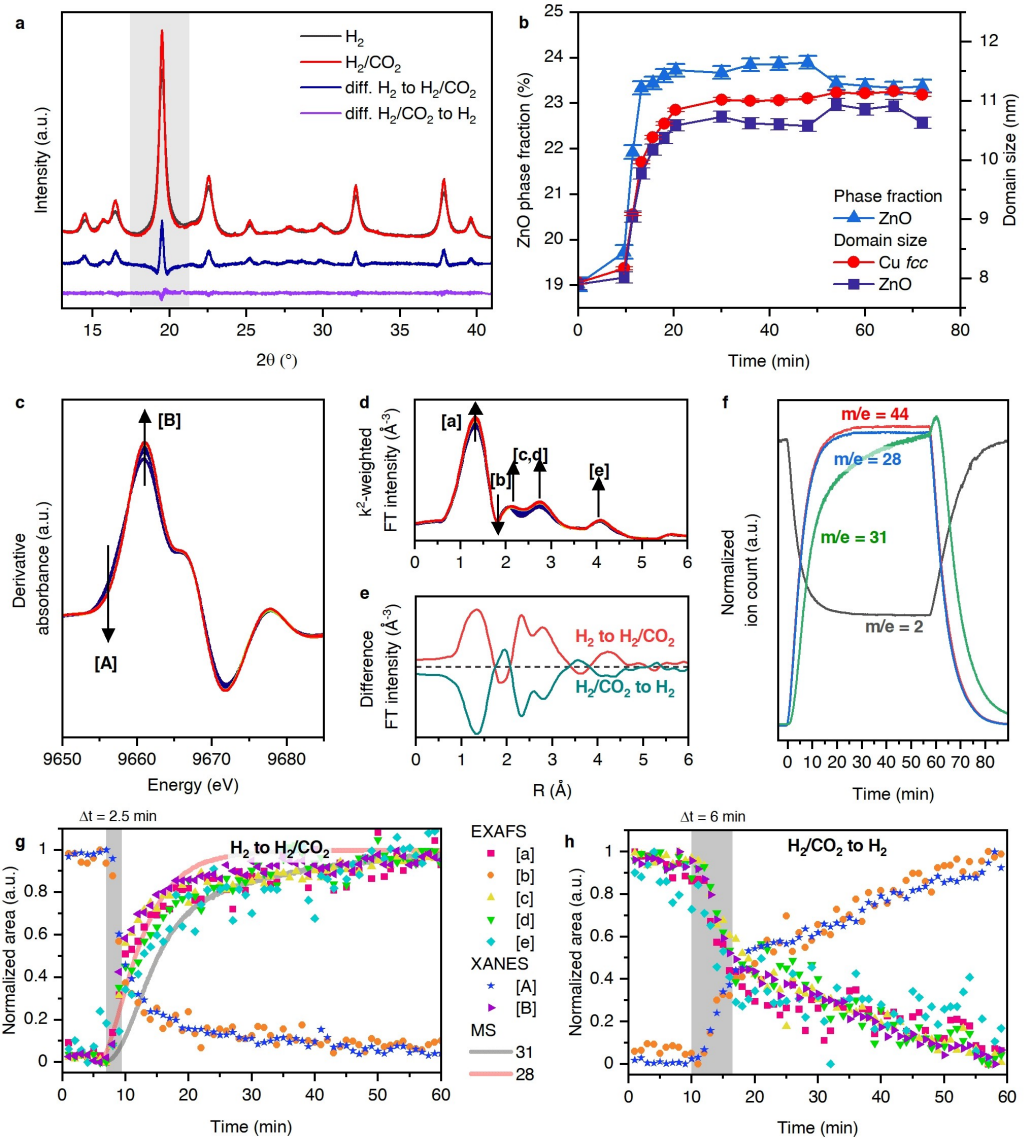
Time‐resolved HR‐XRD derived at 15 bar pressure and at 533 K for a reactive switch from a hydrogen feed to a catalytic (3 H_2_:CO_2_): a) Exemplary diffraction obtained from the CZA system under hydrogen, and after 30 min under 3 H_2_:CO_2_. The difference XRD pattern between these two points in time. And for the reverse gas switch from H_2_/CO_2_ to H_2_. b) Evolution of the ZnO phase fraction and the domain sizes of Cu *fcc* and ZnO, determined by Rietveld refinement, during the switch from H_2_ to H_2_/CO_2_. c)–h) Isobaric (12 bar) and isothermal (533 K) reactive switching to and from a catalytic reaction mixture (3 H_2_:CO_2_) to a purely reductive one (H_2_) over the CZA sample as viewed from the perspective of Zn K‐edge XAS. c First derivatives of the Zn K‐edge XANES during the switch to reaction mixture, wherein we identify changes in the features [A] and [B]. d) FT of the Zn K‐edge EXAFS during the same switch (temporal evolution is colour coded from blue to red). e) Difference spectra of the FT Zn K‐edge EXAFS between hydrogen and after 30 min in H_2_/CO_2_ and difference of the reverse switch from H_2_/CO_2_ to H_2_ after 30 min in H_2_. f) Evolution of the *m*/*e*=2 (H_2_), 28 (CO/CO_2_), 31 (CH_3_OH), and 44 (CO_2_) online mass spectrum during the catalytic switches. g) Evolution of the peak areas determined by Gaussian peak fitting of the XANES derivative ([A] and [B]) and the FT of the EXAFS ([a]–[e]) during the switch from H_2_ to H_2_/CO_2_ and for the reverse switch (panel h).

After following the activation of the CZA precursor in hydrogen, we now consider what happens to the CZA catalyst when the feed is isothermally and isobarically switched between a catalytic feedstock (3 H_2_:CO_2_) and hydrogen. Figure 3a shows exemplary XRD patterns after 600 seconds, subsequent to the switch to and from the catalytic feedstock, along with the difference lines. The patterns show that the activated CZA responds to the change in reactive environment. All visible Bragg reflections sharpen after the switch to catalytic conditions. Within the timescale of the experiment, these changes are not reversed by the switch to pure hydrogen as the difference for this latter switch yields a flat line (Figure 3a). Explicit analysis of the XRD (Figure 3b) quantifies the change in the average domain sizes of both the zinc oxide and the *fcc* metallic phase (from ca. 8 nm to ca. 11 nm in each case) induced by the exposure to carbon dioxide and hydrogen. Phase fraction quantification showed an increase in the Bragg diffraction visible zinc oxide phase (from 19 % to 23 %). As previously,^[42]^ we have also investigated the same switching events at 15 bar using XAS. In the Cu K‐edge XANES minute changes occur (Supplementary Figure S6), which mainly correspond to a shift of the XANES to lower energy, which can be related to the transformation of any CuZn alloy present to metallic copper.^[29]^ The derivative of the Zn K‐edge XANES (Figure 3c) and the EXAFS (Figure 3d) indicate that the zinc structure also responds to the reactive switching. The difference spectra of the FT‐EXAFS for the switch to and from catalytic conditions (Figure 3e) reveal a fully reversible process in the XAS. The Gaussian peak analysis of the XANES and EXAFS (Figure 3g and h, Supplementary Figure S7) shows that all features besides [C] reversibly respond to changed conditions. In contrast to the complex decoupled behaviour in the pre‐treatment procedure (Figure 1), during the catalytic switches, there is a highly synchronized evolution of the peaks. As soon as carbon dioxide is introduced into the reactor, there is steep decrease of the features associated to the reduced zinc [A] and CuZn alloy (Figure 3g). Conversely, all features associated to the zinc oxide structure ([B], [a], [c]–[e]) increase. The decrease and increase of these features resemble an exponential function. For the switch back to hydrogen, however, the evolution is composed of a two‐step process (Figure 3h). The initial fast process appears to stop around 50 % of the total signal change. The time required to reach 50 % of the final transformation is 2.5 min in the case of switching to carbon dioxide and hydrogen and around 6 min for back transformation under pure hydrogen. During this process of the switch to catalytic conditions the evolution of the mass signal of methanol (*m*/*z*=31) increases with a slight delay behind the structural transformations and the evolution of the carbon dioxide and monoxide associated mass 28 (Figure 3f).

**Scheme 1:**
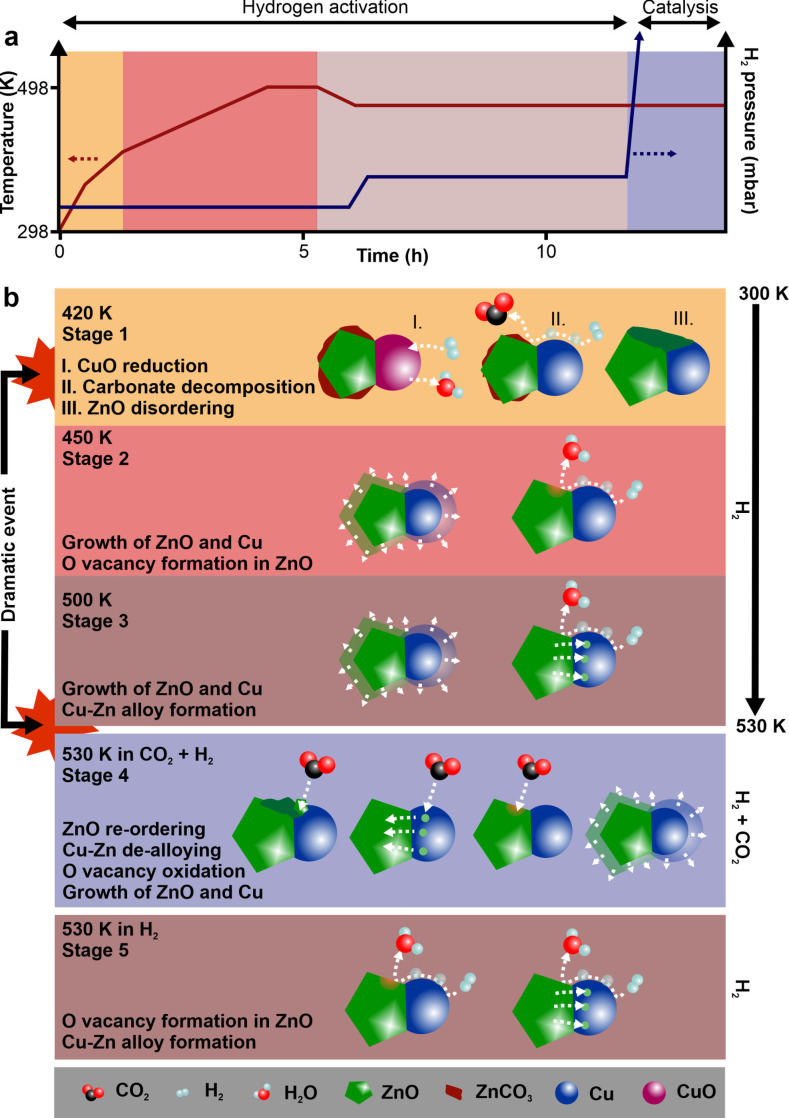
a) Typical reported industrial activation protocol.[^33^, ^34^] b) Genesis and transformation of the CZA precursor to the working catalyst.

In the industrial activation protocols for CZA, two holding points generally define the procedure: an isothermal step within the temperature‐programmed reduction; and, a slow subsequent transition from purely reductive conditions to reaction mixture.[^33^, ^34^] The holding point during the reduction is defined by a relatively fast heating to around 373 K in diluted hydrogen (*p*(H_2_)<200 mbar (Scheme 1a). The temperature is then increased at much lower rates (<0.5 K min^−1^). When reaching the desired temperature of 530 K, the pressure is slowly increased. For the switch to catalytic conditions, the temperature is often decreased to below 480 K, the transition performed, and the temperature increased under reaction conditions again. The combined study of XAS and XRD during the catalyst start‐up procedure allows us to understand why this specific protocol exists. We identify the complex multi‐dimensional restructuring of the zinc oxide phase of the CZA catalyst (Stages indicated in Scheme 1). The precursor is composed of copper(II) oxide, zinc oxide, and zinc carbonate. During activation in hydrogen, it undergoes its first major transformation at around 420 K (depending on the hydrogen pressure) when copper(II) oxide reduces to metallic copper (Stage 1). This reduction process triggers multiple fast transformations within the zinc oxide phase. The sudden presence of metallic copper surfaces then allows a new type of interaction of hydrogen with the catalyst. In the presence of metallic copper, hydrogen dissociates more easily and can transfer from there as atomic hydrogen onto and into the zinc oxide phase via hydrogen spillover.[^43^, ^44^] This spilt‐over hydrogen can further react with the zinc phases to, for example, promote the decomposition of zinc carbonates to yield carbon dioxide.^[29]^ The Bragg diffraction visible zinc oxide phase starts to grow (from ca. 3.5 nm) while the reduced copper phase initially remains constant in size. When the zinc oxide phase reaches the size of the larger copper crystals (5 nm), the copper also starts to grow. Both, copper and zinc oxide then grow at the same monotonic rate, as the temperature increases, to around 7.5 nm. However, at the same time, a substantial fraction of the crystalline zinc oxide becomes invisible to Bragg diffraction. This implies that part of the zinc, as a result of the reduction of the copper(II) oxide and formation of a metallic *fcc* phase, transforms into a disordered phase,^[45]^ or is consumed into a CuZn alloy. The EXAFS investigation revealed that at this temperature at most only a very small fraction of the zinc participates in the alloy formation (<5 %) which cannot account for the observed loss of ≈50 %, and therefore strongly suggests that disordering of the zinc oxide is the main event occurring. The formation of small zinc oxide patches on‐top of the copper nanoparticles is well established,[^19^, ^45^] reminiscent of classical SMSI formation.^[46]^ The disappearance of crystalline zinc oxide may be directly correlated to the formation of such overlayers. Consideration of the XAS (Figure 1) shows that around 450 K (Stage 2), a reduction of the zinc oxide occurs initially via the formation of oxygen vacancies as a result of the newly formed copper‐zinc oxide interfaces. These new interfaces induce the formation of Schottky^[26]^ and oxygen vacancy[^47^, ^48^] defects, and these dominate the low binding energy feature [A] in the XANES in the temperature range ca. 400–500 K. At higher pressures, and especially temperatures above 500 K (Stage 3), the subsequent increase in intensity of this feature, is due to the consumption of an increasing proportion of the zinc oxide and its incorporation into a growing CuZn phase. We also observe that the growth of the *fcc* copper phase is accompanied by an expansion of the lattice parameter. It grows linear with temperature, but does not attain the expected bulk copper lattice constant (Figure 2c).[^49^, ^50^] With XAS, an increased ordering of the zinc oxide structure (feature [C], [d], and [e]) was also observed, which is revealed by XRD to be due to crystal growth. This growth apparently dominated the XAS data. Further parameterization (Supplementary Figure S8), based on refinement of XRD patterns, reveals that the lattice parameter c of *hcp* zinc oxide deviates from the bulk (5.17 vs. 5.20 Å) and that this parameter decreases when copper oxide is reduced. The lattice parameters a and b, however, remain close to the bulk expectation (3.24 vs. 3.25 Å) and do not change significantly. This may be connected to the decoupled behaviour of the EXAFS features [a] and [c] to the group [C], [d], and [e].

Even though there is no a priori reason, industrial activation protocols do not exceed 530 K—the temperature of catalytic operation. Looking at our data (grey bar in Figure 1d–l), it appears that this temperature window used for activation is the temperature at which the specific ordering in the zinc oxide structure is maximized as well as the amount of vacancies formed, while the amount of CuZn alloy formation is still minimal. Beyond 530 K, this order collapses. This could indeed be the reason, for the maximum reduction temperature in the protocols. At higher temperatures, more CuZn alloy is formed which is an unwanted state,[^18^, ^42^, ^51^] and as soon as the zinc oxide structure collapses, potentially also a collapse of the overall high‐surface area could occur.

The application of conditions of carbon dioxide hydrogenation marks the second dramatic event which further modifies the structure of the CZA (Stage 4). The domain sizes of both the metallic *fcc* phase and the zinc oxide grow in response to the new conditions within the first 10 min. This may have multiple origins, as water starts to be produced by the reaction and an exothermic catalytic process starts to occur at the catalyst surface. After this time, the crystal growth comes to an end. At the same time, a fraction of the disordered zinc oxide appears to transform again to a well‐ordered zinc oxide. The oxidation potential of carbon dioxide leads to a re‐ordering of the zinc oxide which was implied to have become disordered under pure hydrogen subsequent to the formation of the metallic copper phase. However, the fraction of Bragg diffraction visible zinc oxide remains much smaller than the starting fraction of 37 % in the CZA precursor prior to activation. The mass spectrum recorded during the switch (Figure 3f) shows a delayed evolution of the methanol‐related mass 31. This delay potentially means that the structure, which is more selective towards methanol needs to be formed first. However, a retardation of methanol in the gas stream can also be due re‐adsorption of methanol on the catalyst and a consequent delay in the establishment of steady state conditions. When the catalyst is again exposed to pure hydrogen (Stage 5) no further changes occur on the level of particle size. Yet, on the atomic scale (XAS), pure hydrogen re‐establishes oxygen vacancies, a small fraction of CuZn alloy, and, on this scale, the zinc oxide ordering decreases, most likely due to the formation of vacancies within the zinc oxide lattice. Thus, there is a reversible redox process within the zinc oxide. This redox cycle appears closely related to the redox cycle of carbon dioxide hydrogenation. The formation of methanol presumably occurs via a redox mechanism involving zinc.[^48^, ^51^] The oxidation of the reduced zinc occurs as a single process which can be described with an exponential function. The re‐reduction and formation of these species however occurs by a different kinetic, namely a two‐step process, an initial fast and a subsequent slower process. In the redox process that we are observing, the first and faster reduction process is still substantially slower than its oxidation counterpart. Given these observations, the reduction of zinc oxide may be a rate‐limiting step to the regeneration of the materials within the catalytic cycle. This would be consistent with the close to zero rate order in carbon dioxide for CZA catalysts in methanol synthesis, while the rate order in hydrogen is generally reported to be >0.5.^[13]^

## Conclusion

The synergism of the components in CZA and relation to catalytic performance is well‐established. We show that this synergism is already reflected in the copper–zinc structure relations which appear during the genesis of the working state catalyst. This transformation from precursor to active catalyst is defined by two dramatic events. These transformations are highly correlated within the copper and zinc phases. The copper has a determining effect on the final structure of the zinc oxide and vice versa. We can now pinpoint during which stages in the catalyst activation this deterministic synergism, which exists between the copper and zinc phases, has the most drastic effects. To define and comprehend such events during the start‐up protocol remains crucial to guide the catalyst precursor in a controlled manner in order to yield an active and long‐lasting catalyst (several years).^[32]^ In fact, the activation protocol which is the current standard of CZA catalysts is optimized for methanol synthesis from carbon monoxide containing feeds. For the emerging use of pure carbon dioxide as carbon source, the optimization of the activation protocol may be still pending, since the two conditions may require different active structures.^[52]^ Any heterogeneous catalyst and catalytic process should be investigated for its transformation from precursor to active catalyst to steer treatment protocols to the optimum structure.

## Conflict of interest

The authors declare no conflict of interest.

1

## Supporting information

As a service to our authors and readers, this journal provides supporting information supplied by the authors. Such materials are peer reviewed and may be re‐organized for online delivery, but are not copy‐edited or typeset. Technical support issues arising from supporting information (other than missing files) should be addressed to the authors.

Supporting Information

## Data Availability

Additional data is presented in the Supporting Information and further raw data is stored in the public data repository ETH Zurich's Research Collection under https://doi.org/10.3929/ethz‐b‐000534344 .
