# Supplementary material for: Drastic Events and Gradual Change Define the Structure of an Active Copper‐Zinc‐Alumina Catalyst for Methanol Synthesis
Source: Angew Chem Int Ed Engl. 2022 Mar 2;61(15):e202200301. doi: 10.1002/anie.202200301 (PMC9314061; doi:10.1002/anie.202200301)
Supplement: Supplementary file 1 — Supporting Information [file ANIE-61-0-s001.pdf]

Angewandte Chemie
Eine Zeitschrift der Gesellschaft Deutscher Chemiker

Supporting Information

Drastic Events and Gradual Change Define the Structure of an Active Copper-Zinc-Alumina Catalyst for Methanol Synthesis

*A. Beck, M. A. Newton, M. Zabilskiy, P. Rzepka, M. G. Willinger, J. A. van Bokhoven**

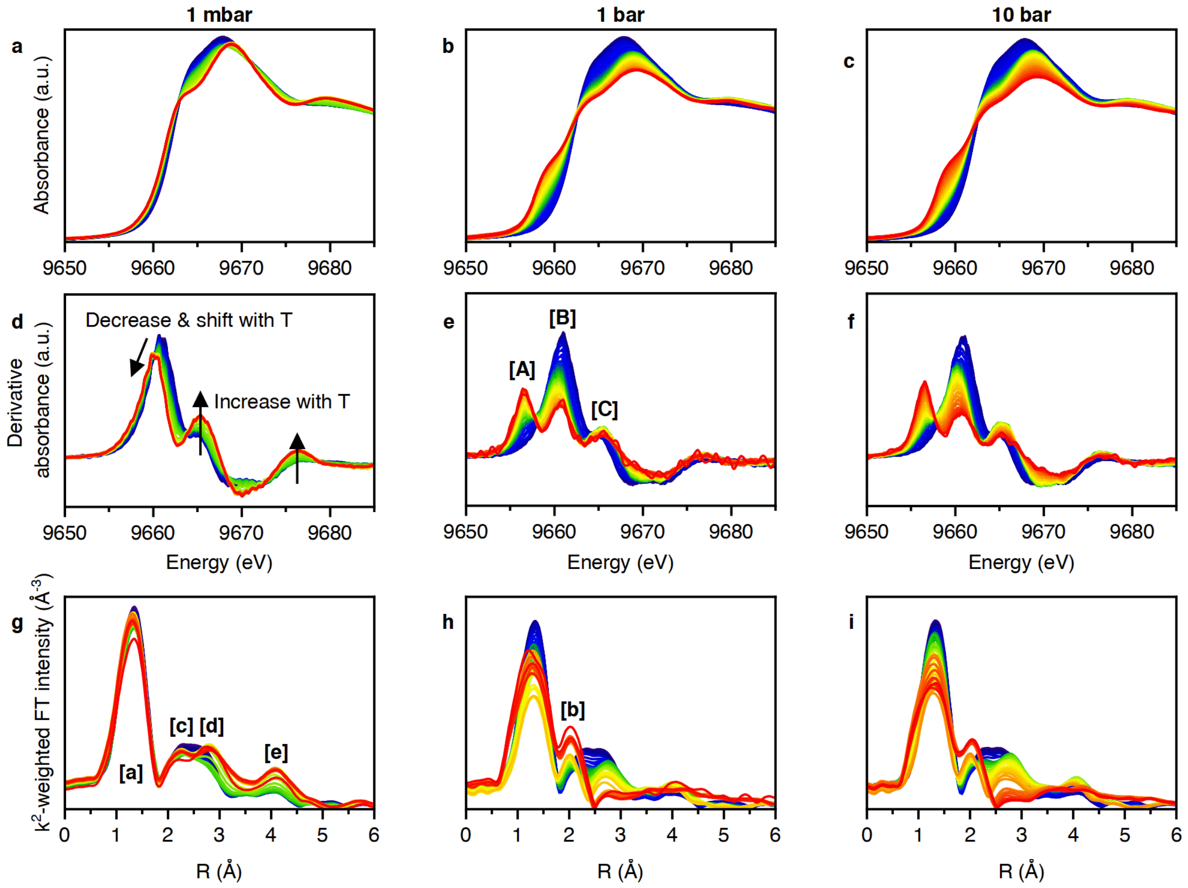

Supplementary Figure S1: Zn K-edge XANES (a-c) during the H_2-TPR at 1 mbar, 1 bar, and 10 bar partial pressure of hydrogen. (d-f) The respective first derivatives of the XANES region. (g-i) The non-phase corrected k^2-weighted FT-EXAFS of the Zn K-edge. XANES derivative peaks [A]-[C] and [a]-[e] are indicated as used in the manuscript.

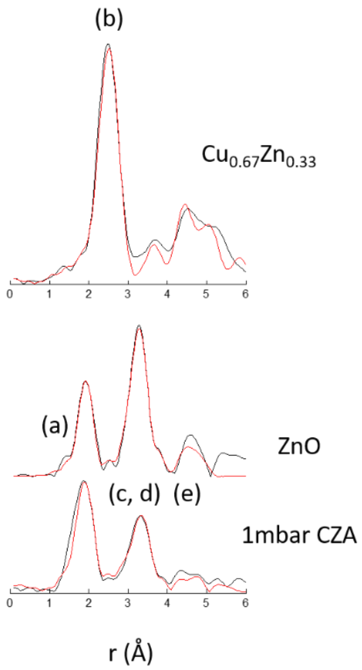

Wurtzite (refined to ZnO standard)

CN	Element	Distance (Å) No phase correction	Distance (Å) Phase corrected	
1	O	1.35	1.99	Zn- O (A)
3	O		1.95	
3	Zn		3.09	Mainly Zn- Zn (C, D)
3	Zn	2.30, 2.76	3.16	
6	Zn		3.28	
1	O		3.69	
6	O		3.75	
3	O		3.87	
3	Zn	4.11	4.51	Zn- Zn (E)
3	Zn		4.64	

Supplementary Figure S2: Association of features present in non-phase corrected and phase corrected Fourier transform representations of Zn K-edge EXAFS on the basis of fitting *hcp* ZnO bulk and $Cu_{0.67}Zn_{0.33}$ foils. For comparison, the FT of Zn K-edge EXAFS due to the CZA sample measured at RT under 1 mbar of H_2 is also given.

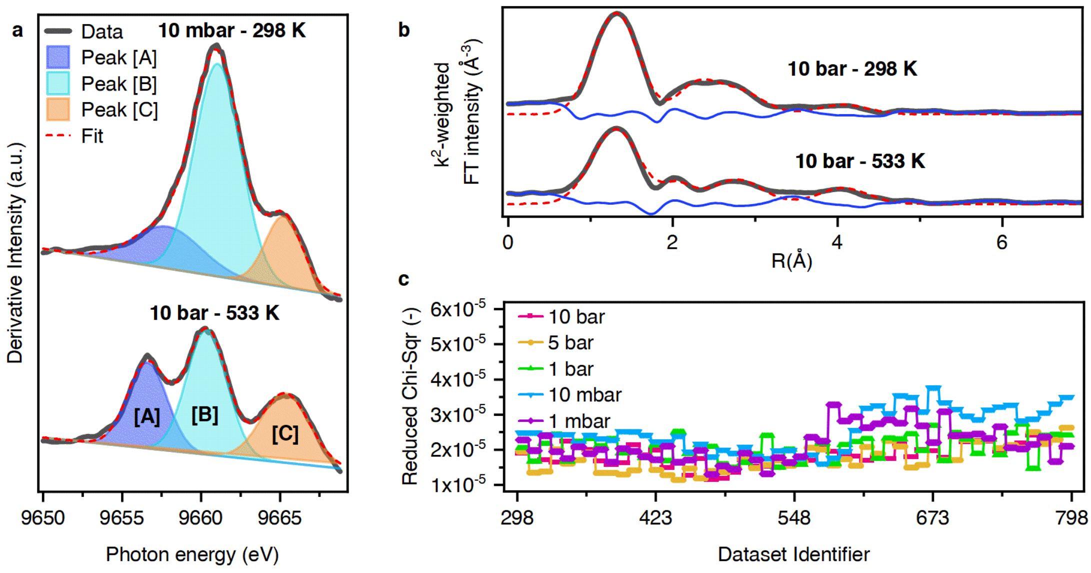

Supplementary Figure S3: a Exemplary fitting results of the derivative Zn K-edge XANES and b FT-EXAFS using three Gaussian component during the TPR and c the respective Chi square values of the fitting.

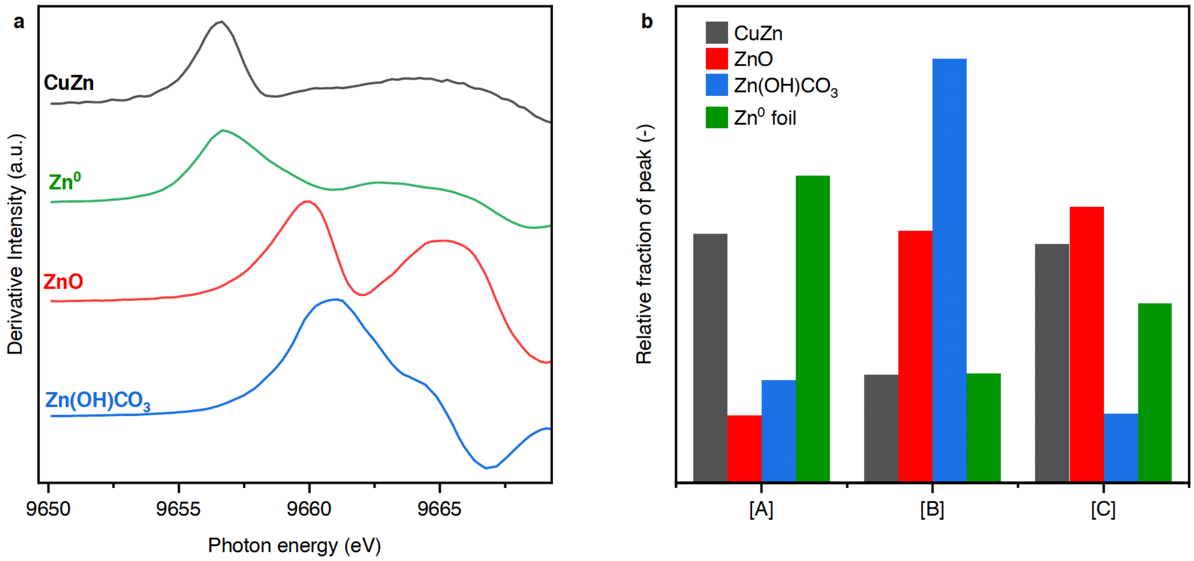

Supplementary Figure S4: Zn K-edge XANES derivatives of measured bulk standard references (**a**). The relative fraction (**b**) of each compound to the peaks [A], [B], and [C].

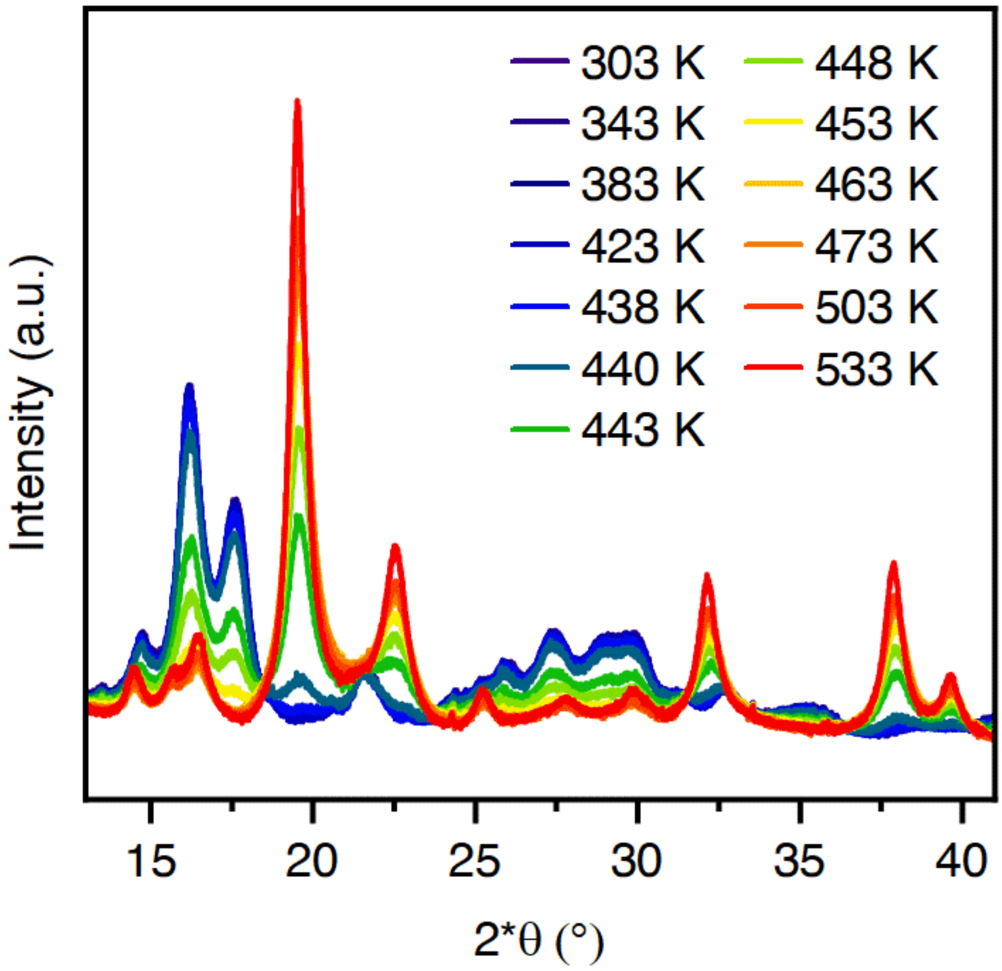

Supplementary Figure S5: Evolution of the XRD pattern during temperature-programmed reduction.

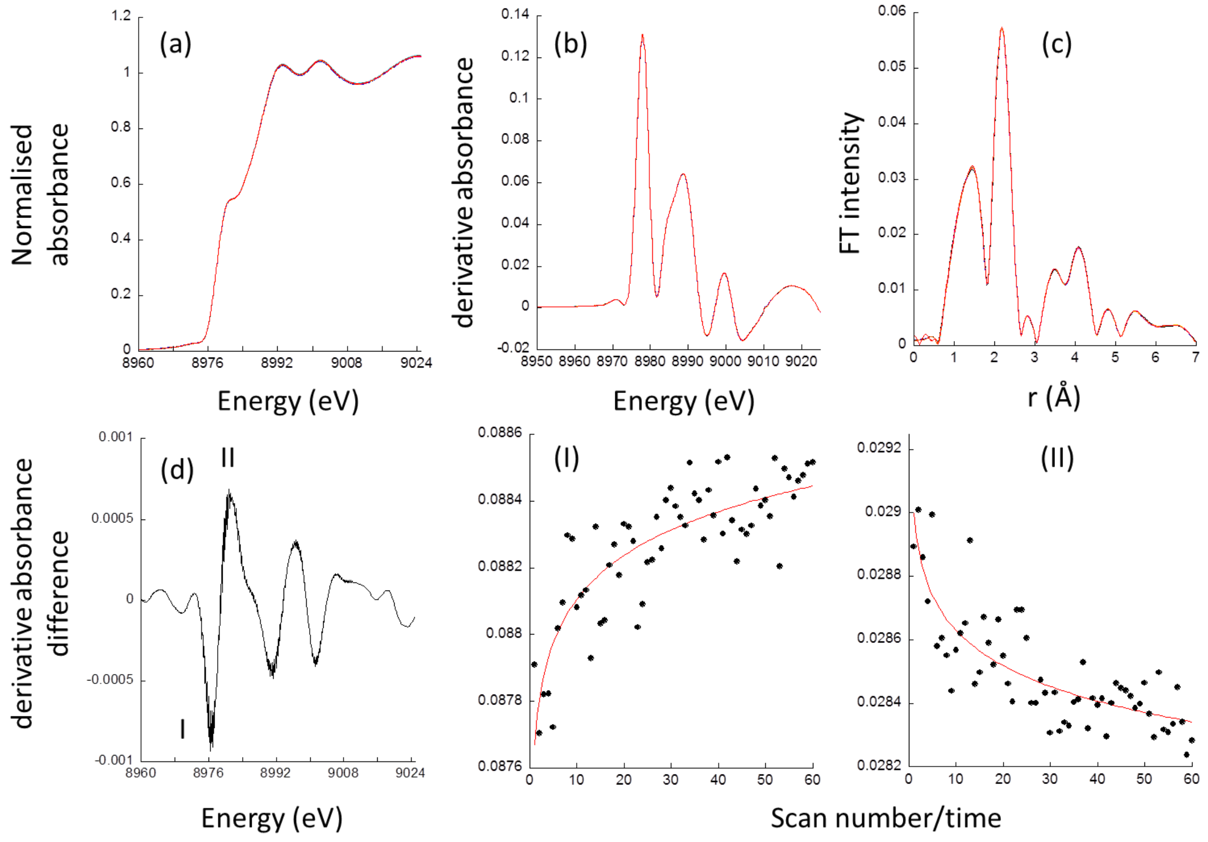

Supplementary Figure S6: Examples of Cu K-edge (a) normalised XANES, (b) the derivative of the Cu K-edge XANES, and (c) the Fourier transform of the Cu K-edge EXAFS. Each graph shows the spectrum obtained initially under 15 bar hydrogen at 533K (black) along with that obtained subsequent to a reactive switch to a $3H_2:CO_2$ flow at the end of the measurement (red) along with the residuals which remain after a simple difference is made between these two experimental points (red – black).

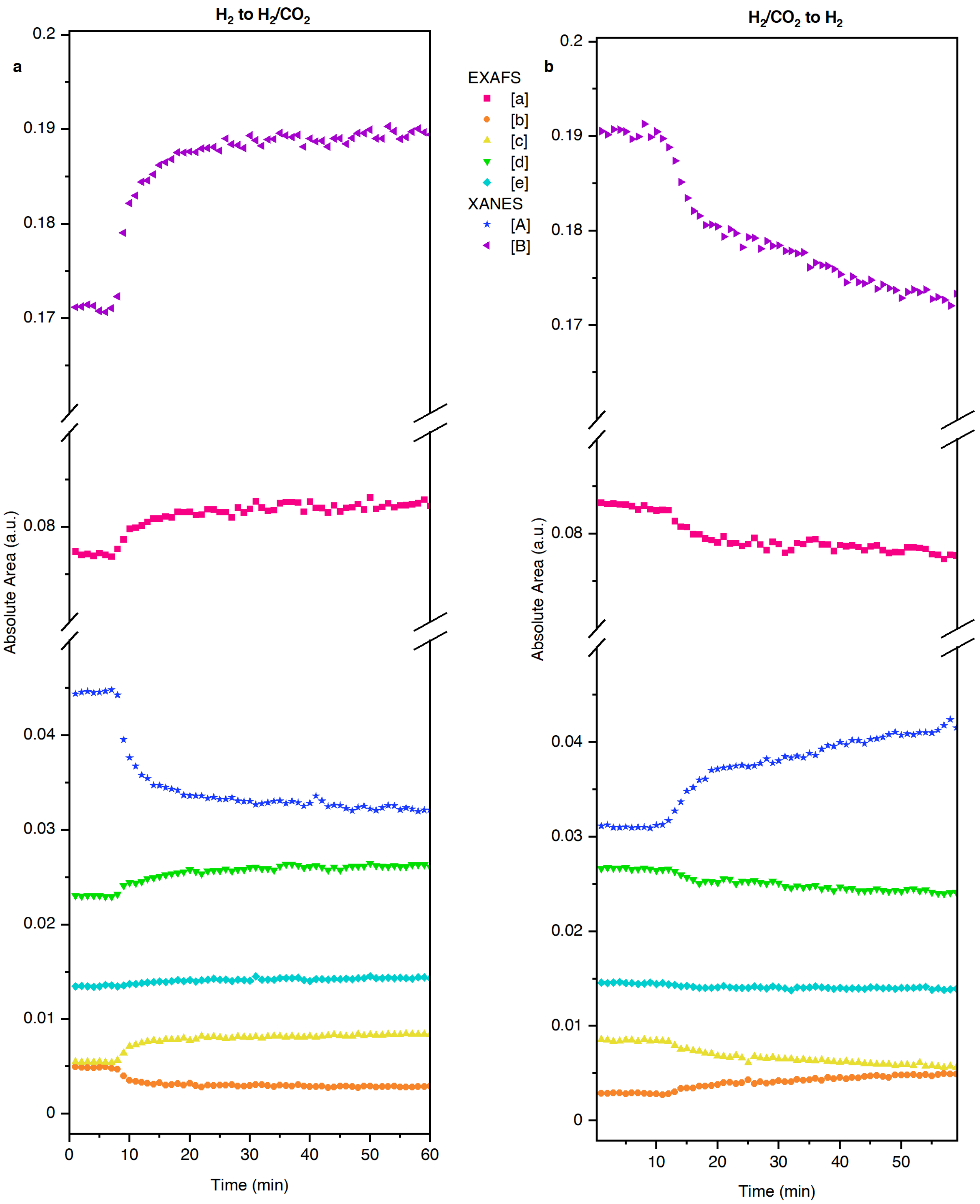

Supplementary Figure S7: Absolute area evolution of the various peaks analyzed in the XANES and EXAFS during the catalytic switches from and to carbon dioxide and hydrogen from pure hydrogen.

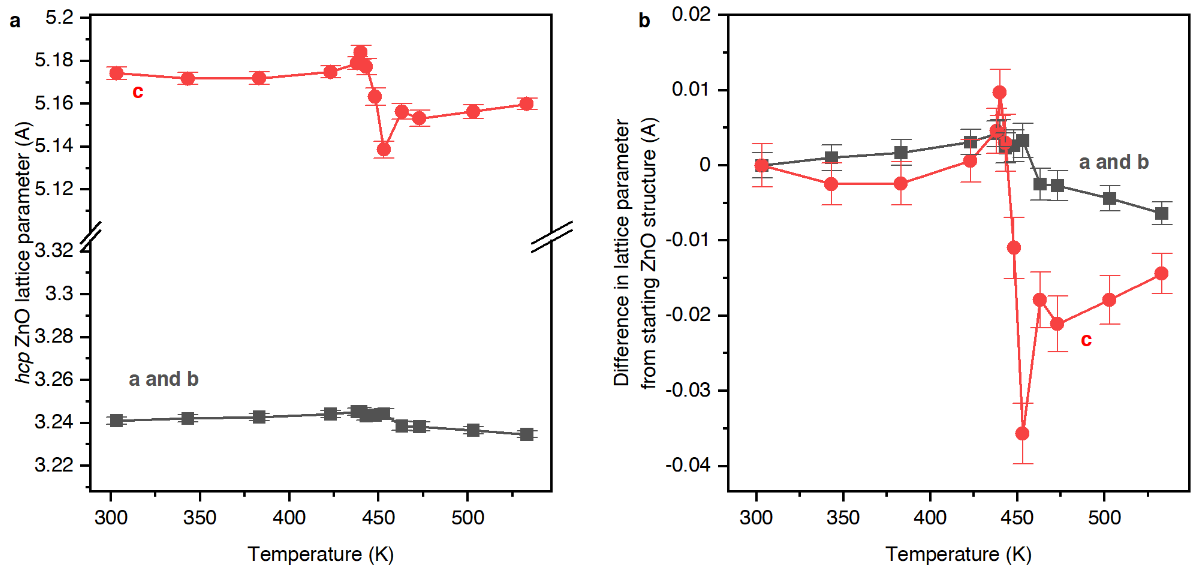

Supplementary Figure S8: Evolution of the hcp ZnO lattice parameters as a function of temperature during TPR (a). (b) shows the difference of the lattice parameter with respect to the initial starting value at room temperature.
